# Maternal One-Carbon Nutrient Intake and Risk of Being Overweight or Obese in Their Offspring—A Transgenerational Prospective Cohort Study

**DOI:** 10.3390/nu16081210

**Published:** 2024-04-19

**Authors:** Leonie H. Bogl, Susanne Strohmaier, Frank B. Hu, Walter C. Willett, A. Heather Eliassen, Jaime E. Hart, Qi Sun, Jorge E. Chavarro, Alison E. Field, Eva S. Schernhammer

**Affiliations:** 1 Department of Epidemiology, Center for Public Health, Medical University of Vienna, 1090 Wien, Austria; leonie-helen.bogl@bfh.ch (L.H.B.); susanne.strohmaier@meduniwien.ac.at (S.S.); 2 School of Health Professions, Bern University of Applied Sciences, 3012 Bern, Switzerland; 3 Department of Nutrition, Harvard T.H. Chan School of Public Health, Boston, MA 02115, USAjchavarr@hsph.harvard.edu (J.E.C.); 4 Department of Epidemiology, Harvard T.H. Chan School of Public Health, Boston, MA 02115, USA; 5 Channing Division of Network Medicine, Department of Medicine, Brigham and Women’s Hospital, Boston, MA 02115, USA; rejch@channing.harvard.edu; 6 Department of Environmental Health, Harvard T.H. Chan School of Public Health, Boston, MA 02115, USA; 7 Department of Epidemiology, Brown University, Providence, RI 02903, USA

**Keywords:** maternal diet, perinatal programming, childhood overweight, pregnancy diet, choline, phosphatidylcholine

## Abstract

We aimed to investigate the associations between maternal intake of folate, vitamin B12, B6, B2, methionine, choline, phosphatidylcholine and betaine during the period surrounding pregnancy and offspring weight outcomes from birth to early adulthood. These associations were examined among 2454 mother–child pairs from the Nurses’ Health Study II and Growing Up Today Study. Maternal energy-adjusted nutrient intakes were derived from food frequency questionnaires. Birth weight, body size at age 5 and repeated BMI measurements were considered. Overweight/obesity was defined according to the International Obesity Task Force (<18 years) and World Health Organization guidelines (18+ years). Among other estimands, we report relative risks (RRs) for offspring ever being overweight with corresponding 95% confidence intervals across quintiles of dietary factors, with the lowest quintile as the reference. In multivariate-adjusted models, higher maternal intakes of phosphatidylcholine were associated with a higher risk of offspring ever being overweight (RRQ5vsQ1 = 1.16 [1.01–1.33] *p*-trend: 0.003). The association was stronger among offspring born to mothers with high red meat intake (high red meat RRQ5vsQ1 = 1.50 [1.14–1.98], *p*-trend: 0.001; low red meat RRQ5vsQ1 = 1.05 [0.87–1.27], *p*-trend: 0.46; *p*-interaction = 0.13). Future studies confirming the association between a higher maternal phosphatidylcholine intake during pregnancy and offspring risk of being overweight or obese are needed.

## 1. Introduction

The prevalence of obesity in the United States remains high, with about 40% of adults and 18.5% of children and adolescents classified as being obese [1]. Obesity primarily results from prolonged energy intake surpassing energy expenditure, with potential influences from epi(genetic) factors and the gut microbiota affecting individual responses to diet and physical activity [2]. Environmental stimuli in early life can induce enduring metabolic changes, programming fetal susceptibility to adiposity in later life [3].

A well-established example is the adequate intake of folate during early development, in the prevention of neural tube defects [4]. While the underlying mechanisms for early programming are not yet fully elucidated, dietary inputs to the one-carbon metabolic pathway during the periconceptional period may influence adult health-related phenotypes. The one-carbon metabolism is a biochemical process involved in the transfer of single-carbon units (methyl groups) between molecules, making it crucial for DNA synthesis, amino acid metabolism and various cellular functions including methylation reactions and the maintenance of cellular homeostasis. Some nutrients, including folate, vitamin B2, vitamin B12, vitamin B6, betaine, choline and methionine, act as enzymatic cofactors or methyl donors in one-carbon metabolism [5].

Another possible mechanism through which maternal nutrient intake during pregnancy could influence offspring disease risk is the composition of the gut microbiome [6]. The colonic gut microbiota varies by dietary intake of folate and other B vitamins, so that individuals with a low dietary intake of B vitamins involved in one-carbon metabolism have a lower abundance of butyrate-producing gut bacteria and a higher abundance of potentially harmful bacteria [7]. Choline, and its major dietary source phosphatidylcholine, is metabolized by the gut microbiota to trimethylamine which is further oxidized in the liver to the proatherogenic metabolite trimethylamine N-oxide (TMAO). Increased levels of gut-derived TMAO have recently been linked to obesity traits in mice and to type 2 diabetes in multiple clinical and cohort studies [8,9].

Previous research in Wistar rats suggests that excessive maternal intakes of choline [10] and folic acid [11], often facilitated by prenatal vitamins, increase the body weight in the offspring, likely through the programming of appetite in the hypothalamus or due to changes in the gut microbiota. There is a lack of human research supporting the findings observed in animal studies. The existing data predominantly examine the relationship between intake of specific one-carbon nutrients (choline, vitamin B12, folate) and childhood weight outcomes, with results showing considerable inconsistency [12,13,14].

The aim of the present study is therefore to address this research gap by examining the associations of maternal one-carbon nutrient intake (folate, vitamin B12, B6, B2, methionine, choline, phosphatidylcholine and betaine) surrounding pregnancy with the risk of the offspring being overweight or obese. We therefore utilized the ongoing Nurses’ Health Study II (NHSII) and Growing Up Today Study 2 (GUTS2), with a cohort of children of the women participating in NHSII with a long-term follow-up until early adulthood.

## 2. Materials and Methods

### 2.1. Study Population

We utilized information on mothers from the NHSII and their offspring participating in the GUTS2. The ongoing prospective cohort study NHSII was established in 1989 when 116,429 female nurses aged 25–42 years completed a baseline questionnaire about their lifestyle behavior and medical history. Follow-up questionnaires are mailed to the nurses every two years to update the information on potential risk factors and diseases, including a food frequency questionnaire (FFQ) every 4 years since 1991. Details about the NHSII have been described elsewhere [15].

Study participants for GUTS2 were first recruited in 2004, when children born between 1987 and 1995 to NHSII mothers received invitation letters and questionnaires, after obtaining maternal consent. A total of 10,918 children completed the baseline questionnaire and were sent follow-up questionnaires on health, lifestyle and growth indicators in 2006, 2008, 2011, 2013, 2014, 2015, 2016, 2019 and 2021. For the current study, we combined information on maternal diet and covariates from NHSII with information on offspring outcomes from GUTS2. Informed consent was implied by returning the baseline self-administered questionnaire in both cohorts. The study has approval from the Committees on the Use of Human Subjects in Research at the Brigham and Women’s Hospital and the Harvard T.H. Chan School of Public Health (Boston, MA, USA).

### 2.2. Assessment of Maternal Nutrient Intake during the Period Surrounding Pregnancy

Maternal nutrient intake during the period surrounding pregnancy was assessed using the 131-item semiquantitative food-frequency questionnaire (FFQ) in 1991 closest to the considered pregnancy. The mothers were asked how often, on average, they consumed the specified amount of each type of food or beverage during the past 12 months, with 9 possible frequency response categories ranging from ‘never or less than once per month’ to ‘6 or more times per day’.

Nutrient intakes were calculated from the FFQ by multiplying the frequency of intake for each food item or supplement by its nutrient content and then summing across all items. Calculation of total energy intake and average nutrient intake for total folate, vitamin B12, vitamin B6, vitamin B2, methionine, choline, phosphatidylcholine and betaine was based on values obtained from the USDA database on the composition of foods and the database for the choline content of common foods [16] and other sources [17]. Total choline intake was the sum of free choline plus choline from each of the choline-containing compounds (glycerophosphocholine and phosphocholine) and lipid-soluble compounds (i.e., phosphatidylcholine and sphingomyelin [18]). The FFQ has been shown to be a valid instrument for measuring nutrient intakes compared with multiple dietary records, 24 h dietary recalls and biomarkers among women, with mean correlation coefficients of around 0.6 or higher for energy-adjusted B vitamins and choline [19]. Further, higher intakes of dietary choline were related to lower homocysteine concentrations independent of folate and other B vitamins, supporting the validity of choline intake measured by the FFQ [20].

### 2.3. Assessment of Weight Outcomes among Offspring

#### 2.3.1. Primary Outcome

Body mass index (BMI) in the offspring was calculated from self-reported weight and height on each GUTS2 follow-up questionnaire (2004, 2006, 2008, 2011, 2013, 2014, 2015, 2016, 2019 and 2021), i.e., from age 13 onwards. Previous studies on the validity of self-reported weight and height generally found that adolescents and young adults provide valid information [21]. Definitions for normal weight, overweight and obese were based on the age- and sex-specific cutoffs from the International Obesity Task Force for participants aged 18 years and younger [22]. After age 18, definitions were based on the World Health Organization cutoffs (i.e., BMI between 25 and 29.9 kg/m^2^ and BMI ≥ 30 kg/m^2^ for overweight and obese, respectively) [23]. We considered the outcome ‘ever being overweight or obese’ as our primary outcome by defining offspring as cases if they ever fell into the described category at any given time during follow-up [24].

#### 2.3.2. Secondary Outcomes

Information on offspring birth weight was collected through the GUTS2 mothers’ questionnaire in 2009. The maternal recall of offspring birth weight was found to be reproducible and accurate [25]. Information on offspring body size at age 5 was assessed in the GUTS2 baseline questionnaire in 2004. Participants, then aged 10–17, were asked to report their body size at age 5 by selecting one of eight pictograms that most accurately represented their body shape within a range from 1 = most lean to 8 = most obese. For the present analyses, children with a reported body shape larger than the median of the distribution were defined as cases [24].

### 2.4. Assessment of Covariates

Maternal age at birth of the child was calculated based on the birth dates of the mothers and their offspring. Smoking status, physical activity before pregnancy and pre-pregnancy BMI were derived from the 1989 NHSII questionnaire for women who were not pregnant at the time of the questionnaire return. Maternal marital status was also obtained from the 1989 NHSII questionnaire. Total energy intake and other dietary variables were calculated from the FFQ. The husband’s education was assessed in 1999 and was considered a proxy for socioeconomic status. Parity and gestational age at delivery were obtained from the lifetime pregnancy assessment in 2009. If mothers did not return the 2009 questionnaire, a missing indicator was introduced. Children’s sex and age were obtained from the baseline GUTS2 questionnaire in 2004. For detailed covariate categories used in the analyses, see Table 1 and the footnotes of all tables.

### 2.5. Final Analytic Samples for the Analyses

**Figure 1:**
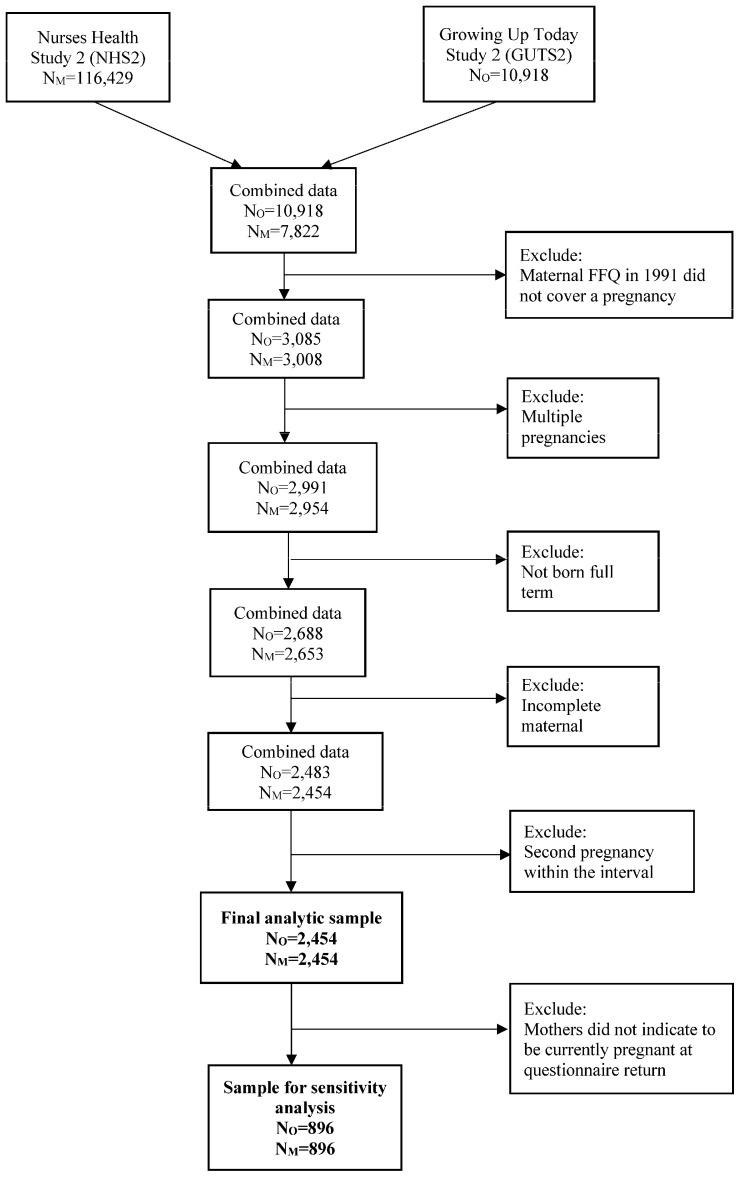
Flowchart depicting exclusion steps to arrive at the final analytic sample for the main analyses and sensitivity analysis. N_O_ denotes the number of offspring; N_M_ denotes the number of mothers.

Children enrolled in GUTS2 (n = 10,918) were born between 1987 and 1995 to n = 7822 mothers enrolled in NHSII (see flowchart in Figure 1). 

In the current study, we included children born surrounding the 1991 diet assessment in our analyses, since maternal diet was assessed for the first time in 1991. We estimated the time of conception based on the children’s date of birth and then selected all mother–child pairs where at least part of the pregnancy was covered by the 12-month period that the FFQ enquired about (n = 3085 children, n = 3008 mothers). We further restricted the sample to singleton births (n = 2991 children, n = 2954 mothers), full term (37–41 weeks of gestation)-born children (n = 2688 children, n = 2653 mothers) and children with complete maternal exposure information (n = 2483 children, n = 2454 mothers). If two pregnancies of the same mother were covered by the 12-month period, we included only the first pregnancy, which left us with a total sample of n = 2454 children, born to n = 2454 mothers, for the analyses focusing on the exposure period surrounding pregnancy. In the sensitivity analysis, we restricted this to n= 896 mother–child pairs where the mothers reported currently being pregnant on the 1991 NHS2 questionnaire. The mean age of children was 12.7 years at baseline in 2004 and 29.4 years at the last follow-up in 2021.

### 2.6. Statistical Analysis

Baseline characteristics are shown for mothers and their children across quintiles of dietary folate and phosphatidylcholine intake. Continuous variables are shown as mean ± standard deviation or median (IQR) and categorical variables are shown as percentages. We calculated relative risks (RRs) and 95% confidence intervals (CIs) for offspring ever being overweight or obese. Due to the high prevalence of the considered outcomes, we used log-binomial models to estimate RRs and approximations based on Poisson models with robust variance estimators in case of convergence problems [26]. Basic models were adjusted for offspring sex and maternal age at birth of the child. The multivariate model was additionally adjusted for potential confounders including maternal pre-pregnancy BMI, smoking status and physical activity, as well as marital status, total energy intake, parity, partner’s education, servings of sugar sweetened beverages, refined grains and quintiles of coffee, trans fat and the ratio of polyunsaturated to saturated fat.

We performed additional stratified analyses by the major food sources of phosphatidylcholine (i.e., eggs, red meat and fish; low or high intakes based on the median as the cutoff) and assessed the interaction by testing the significance of the multiplicative interaction term in our models [9,27]. We also investigated effect modification by maternal pre-pregnancy BMI (normal weight vs. mothers who are overweight or obese) [28].

In sensitivity analyses, we further mutually adjusted for major nutrients involved in the one carbon metabolism simultaneously, including folate, vitamin B-12, vitamin B-6 and vitamin B-2, as well as methionine and betaine. The analysis for phosphatidylcholine was additionally adjusted for the non-phosphatidylcholine component of choline (subtracting phosphatidylcholine from total choline). In separate models, we additionally adjusted for the major food sources of phosphatidylcholine—eggs, fish and red meat.

In secondary analyses, we estimated mean differences (MDs) and 95% CIs in offspring birth weight across quintiles of dietary factors considering the lowest quintile as the reference category and we calculated RRs and 95% CIs for offspring reporting a larger than median body size at age 5.

To ensure that the diet information pertains to the period of pregnancy (and not any period after pregnancy), the associations between nutrient intakes and the risk of the offspring being overweight or obese were also estimated among the subset of mother–child pairs where the mothers reported to be pregnant at the time of filling out the questionnaire.

In all analyses, we introduced missingness indicators for covariates with missing values. Sample sizes may differ by analysis due to the availability of outcome measures. All statistical tests were two sided and were considered statistically significant at *p* < 0.05. Analyses were conducted using SAS version 9.4 (SAS Institute, Cary, NC, USA).

## 3. Results

Descriptive characteristics of the 2454 mother–child pairs included in our analyses show that mothers with higher intakes of folate had a lower BMI before pregnancy and tended to have healthier lifestyle than mothers with lower intakes of folate. However, the opposite trend was seen for mothers with higher intakes of phosphatidylcholine (Table 1). In addition, mothers in the highest quintiles of folate intake were more likely to have partners with a graduate degree but less likely to be married, while mothers in the highest quintiles of phosphatidylcholine intake were less likely to have partners with a graduate degree compared to the lowest quintile. The analysis included slightly more female (53.3%) than male (46.7%) offspring.

**Table 1:** Maternal and offspring characteristics by quintiles of dietary folate and phosphatidylcholine intake during the period surrounding pregnancy for 2454 mothers and full-term-born children between 1990 and 1992, enrolled in the Growing Up Today Study.

	Quintile of Dietary Folate Intake			Quintile of Dietary Phosphatidylcholine Intake		
	Q1	Q3	Q5	Q1	Q3	Q5
Number of mother–child pairs	489	490	491	490	492	490
*Maternal characteristics before pregnancy*						
Body mass index (kg/m^2^) before pregnancy ^a^	23.3 ± 4.4	22.5 ± 3.8	22.7 ± 3.8	22.0 ± 3.2	22.8 ± 4.2	23.5 ± 4.3
Parity before included pregnancy, % ^b^						
Nulliparous	15.4	20.9	27.5	25.8	21.6	16.6
One previous pregnancy	30.9	34.0	30.6	30.9	29.3	33.2
Two previous pregnancies	28.1	24.2	22.4	22.0	29.8	23.7
Three previous pregnancies	25.7	20.9	19.6	21.3	19.3	26.6
Smoking history before pregnancy, %						
Never	68.7	71.9	77.3	76.2	74.8	68.4
Past	21.7	22.8	18.5	18.3	17.4	24.4
Current	9.6	5.3	4.2	5.5	7.8	7.1
*Maternal characteristics during pregnancy*						
Maternal age at birth of the child ^a^, y	32.5 ± 3.8	32.8 ± 3.5	33.0 ± 3.7	32.9 ± 3.6	32.7 ± 3.5	33.1 ± 3.8
Partner’s education, % ^b^						
High school degree	39.5	31.2	31.5	28.3	34.6	33.3
College degree	29.6	34.7	33.4	32.8	34.6	34.6
Graduate school degree	30.9	34.1	35.1	38.9	30.7	32.0
Married in 1989, % ^b^	91.8	93.7	89.8	90.4	92.9	90.4
Total energy intake ^c^ (kcal/d)	1846 (1473; 2225)	1973 (1706; 2326)	1717 (1435; 1957)	1884 (1535; 2728)	1970 (1665; 2316)	1864 (1525; 2264)
Alcohol ^c^ (g/d)	0.9 (0; 2.9)	0.9 (0; 2.8)	0.0 (0; 1.9)	0.0 (0; 2.6)	0.9 (0; 2.7)	0.0 (0; 2.0)
Energy-adjusted trans fat ^c^ (g/d)	3.3 (2.6; 4.1)	2.9 (2.3; 3.6)	2.8 (2.2; 3.5)	2.8 (2.2; 3.8)	2.9 (2.3; 3.7)	2.9 (2.3; 3.7)
Coffee ^c^ (cups/d)	0.4 (0; 2.5)	0.4 (0; 2.0)	0.1 (0; 1.0)	0.1 (0; 1.0)	0.4 (0; 1.4)	0.5 (0; 2.0)
Sugar-sweetened beverages ^c^ (servings/d)	0.6 (0.1; 1.3)	0.4 (0.1; 1.0)	0.2 (0.1; 0.9)	0.4 (0.1; 1.0)	0.4 (0.1; 1.0)	0.5 (0.1; 1.1)
Artifically-sweetened beverages ^c^ (servings/d)	0.1 (0; 0.4)	0.1 (0; 0.5)	0.1 (0; 0.4)	0.2 (0.1; 0.9)	0.1 (0.0; 0.6)	0.1 (0.0; 0.3)
Refined grains ^c^ (servings/d)	1.2 (0.8; 1.8)	1.3 (0.8; 1.9)	1.1 (0.7; 1.5)	1.2 (0.7; 1.9)	1.2 (0.8; 1.7)	1.2 (0.8; 1.8)
Ratio of polyunsaturated to saturated fat ^c^	0.5 (0.4; 0.5)	0.5 (0.4; 0.6)	0.5 (0.4; 0.6)	0.5 (0.4; 0.6)	0.5 (0.4; 0.6)	0.5 (0.4; 0.6)
Physical activity, min/week, %						
0	23.7	14.2	17.4	17.7	16.3	17.3
1–149	43.2	40.1	39.9	39.0	43.9	44.9
150–299	16.3	20.0	21.4	21.7	18.1	18.2
≥300	16.8	25.7	21.2	21.5	21.6	19.6
*Offspring characteristics*						
Offspring gender, %						
Male	47.8	45.7	46.0	47.4	45.9	45.5
Female	52.2	54.3	54.0	52.6	54.1	54.5
Offspring age at GUTSII baseline 2004, y	13.4 ± 0.6	13.2 ± 0.5	13.1 ± 0.4	13.2 ± 0.5	13.2 ± 0.5	13.2 ± 0.6

^a^ Mean ± SD (all such values); ^b^ Percentages are of non-missing values (all such values); ^c^ Median (IQR).

In multivariable analyses, we observed that higher total phosphatidylcholine intakes were associated with a higher risk of offspring every being overweight or obese (RRQ5vsQ1 = 1.16 [1.01–1.33] *p*-trend: 0.003) (Table 2). When we further adjusted for folate, vitamin B-12, vitamin B-6, vitamin B-2, methionine and betaine, the association with higher maternal phosphatidylcholine intake and offspring risk of ever being overweight or obese remained significant (*p*-trend = 0.002). The association persisted even after additionally adjusting for the non-phosphatidylcholine components of choline (*p*-trend = 0.004). Likewise, when additionally adjusting the model for all major food sources, higher intakes of phosphatidylcholine remained significantly associated with a higher risk of being overweight or obese (*p*-trend = 0.04).

Associations between maternal phosphatidylcholine intakes and offspring weight outcomes did not differ between normal-weight mothers or mothers who were overweight or obese (*p*-interaction > 0.05). None of the other one-carbon nutrients we examined were associated with the risk of the offspring ever being overweight or obese (Table 2).

The association of higher dietary phosphatidylcholine intake with offspring risk of being overweight or obese appeared stronger among mothers with a high meat intake (*p*-interaction = 0.13) (Table 3). In the multivariate-adjusted model, the RRs (95% CIs) across quintiles of phosphatidylcholine intakes among mothers with a low red meat intake were as follows: 1.00 (reference), 0.94 (0.78, 1.12), 0.94 (0.79, 1.13), 1.05 (0.88, 1.26) and 1.05 (0.87, 1.27) (*p*-trend: 0.46) and among mothers with a high red meat intake were as follows: 1.00 (reference), 1.23 (0.92, 1.65), 1.45 (1.10, 1.92), 1.58 (1.21, 2.08) and 1.50 (1.14, 1.98) (*p*-trend: 0.001).

Compared to mothers with lower intake, mean birth weight was higher among mothers with a higher intake of vitamin B2 (*p* = 0.02) and choline (*p* = 0.004) in the multivariate models (Supplemental Table S1). The risk of having a larger than median body size at age 5 was similar across quintiles of maternal one-carbon nutrient intakes (Supplemental Table S2).

Supplemental Table S3 shows the association between quintiles of maternal one-carbon nutrient intake and risk of being overweight or obese in the subset of mother–child dyads where the mothers reported to be pregnant at the time of the questionnaire return. The observed patterns are fairly similar to the results in Table 2. However, we no longer observed a significant trend for total phosphatidylcholine (RRQ5vsQ1 = 1.18 [0.95–1.46], *p*-trend = 0.10).

## 4. Discussion

In our study including 2454 mother–child pairs, higher maternal dietary intakes of phosphatidylcholine during the period surrounding pregnancy were associated with a long-term risk of being overweight or obese in the offspring. Effect sizes were similar, although not statistically significant when restricting to mother–child pairs where the mother was pregnant at the time of the questionnaire return. The association between maternal phosphatidylcholine intake and offspring’s risk of being overweight and obese was stronger among mothers consuming a diet high in red meat. Further, we observed that higher maternal dietary intake of choline was associated with greater birth weight among offspring.

Foods containing the highest amounts of dietary phosphatidylcholine, the major dietary source of choline, are of animal origin and include eggs, fish and meat [29]. Choline has been discussed to have potential beneficial and harmful effects. On the one hand, choline is part of the B-complex family and an essential component of membrane phospholipids and a precursor for the biosynthesis of the neurotransmitter acetylcholine [30]. Choline is thought to be of particular importance during prenatal development, as it is involved in neurotransmission and brain development, methyl group donation and gene expression [31]. Studies in rodents demonstrated the protective effect of choline during gestation on neurological function and the performance of offspring during cognitive and behavioral tests [32]. Studies in humans are also suggesting that higher maternal choline intake during pregnancy is associated with better cognitive abilities in the offspring [33]. On the other hand, previous work showed that the gut microbiota can metabolize phosphatidylcholine and l-carnitine to trimethylamine, which is further metabolized in the liver to TMAO [34,35]. Increased plasma levels of TMAO have been associated with an increased risk of a major adverse cardiovascular events [35]. In addition, a higher phosphatidylcholine intake from the diet has been associated with increased all-cause and cardiovascular disease mortality in the US population, independent of traditional risk factors [27]. TMAO and its nutrients’ precursor choline have been further suggested as potential early biomarkers of the metabolic syndrome and studies have shown positive correlations between TMAO and BMI [36] and the presence and severity of non-alcohol fatty liver disease [37]. Among multiple prospective US cohorts, dietary phosphatidylcholine intake was associated with a higher incidence type 2 diabetes in both men and women [9], while in a smaller Finnish cohort, phosphatidylcholine was associated with a lower type 2 diabetes risk among men [38].

Few studies, however, have focused on maternal choline intake or status during pregnancy in relation to offspring weight outcomes. In two large mother–offspring cohorts, maternal circulating choline concentrations during pregnancy were associated with higher offspring BMI z-score, skinfold thicknesses and body fat mass at birth, but not with adiposity in early childhood at age 5 years [12]. Similarly, in the present study, we observed an association between choline intake and higher mean birth weight, but not with body size at age 5. However, we were able to examine long-term outcomes and found that offspring born to mothers with a higher phosphatidylcholine intake during pregnancy had a higher risk of being overweight or obese during young adulthood (until about 30 years), suggesting a potential novel transgenerational pathway of obesity susceptibility that may be mediated by the gut microbiome. However, our findings warrant replication in other cohorts that examine the potential effect of maternal phosphatidylcholine intake on later weight outcomes in offspring. Furthermore, future studies should also involve fathers, as preliminary evidence from animal studies suggests that the paternal diet may also be important in programming offspring health [39]. One study in humans involving 74 fathers who filled in 7-day dietary records found that paternal choline intake was related to increased birth weight of the offspring [40].

Interestingly, we found that phosphatidylcholine intake during pregnancy was related to a risk of offspring being overweight or obese among high red meat consumers but not among low meat consumers. Our findings suggest that other nutritive or non-nutritive components in red meat might potentiate the effect of phosphatidylcholine intake on the risk of offspring being overweight and obese. For example, l-carnitine, an abundant nutrient present in red meat, is metabolized also by the gut microbiota to TMAO and people adhering to the western-style dietary pattern are more likely to have higher plasma concentrations of free choline, l-carnitine and TMAO [36].

Other important nutrients of interest in the present study were other B-vitamins in the one-carbon metabolism pathway, in particular the methyl donor folate. In the current study, total folate intake during pregnancy was not related to offspring birth weight, body size at age 5 or the risk of ever being overweight or obese in later adult life. Published studies in humans examining the relationship between folic acid intake during pregnancy and offspring birth weight and long-term weight outcomes are inconsistent, with some reporting a positive association between maternal folate status and birth weight [41], while others show no relationship [42]. Studies on weight outcomes in infancy are equally inconsistent, with some studies suggesting that maternal folate intake or status increases the risk of obesity in offspring, particularly when combined with low vitamin B12 status or intake [13], but other studies show no effect [43]. In a recent prospective study of 1517 child–parent pairs at the Boston Medical Center, the highest childhood risk of being overweight was observed among children of mothers with obesity with low folate concentrations [44]. A recent systematic review on maternal folate status and obesity and insulin resistance in offspring concluded that data from both animal and human studies are still inconsistent [14].

Some limitations of our study should be acknowledged. The FFQ was not designed or validated for use in pregnancy and was not necessarily administered during pregnancy as we selected mother–child pairs where at least a part of the pregnancy was covered by the 12-month period the FFQ was covering in our main analyses. However, our sensitivity analysis among mother–child pairs where the mother was pregnant at the time of the questionnaire return showed similar effect sizes (albeit not statistically significant). Furthermore, it is known that dietary patterns change little from before to during pregnancy [45]. Our study population was comprised of predominantly white US nurses with higher socioeconomic status and a slightly older age at delivery compared to the general population. Thus, our results might not be generalizable to other ethnic or socioeconomic groups, younger mothers or populations where habitual diets are different from those of Western populations. Given the observational nature of our study, a direct causal link between maternal nutrient intake during pregnancy and offspring risk of being overweight or obese cannot be inferred. We adjusted for a range of maternal factors that could potentially confound our results; however, the possibility of residual confounding still remains. The fact that choline and its derivate TMAO are more common among people adhering to a Western-style diet [36] raises the question of confounding by diet quality. However, we carefully adjusted for a range of nutrient factors, including sugar-sweetened beverages, refined grains, coffee, the ratio of polyunsaturated to saturated fat and trans-fat. Furthermore, in our previous publication on diet quality and weight outcomes in the same cohort, we did not observe an association between maternal diet quality, as indicated by the Alternate Healthy Eating Index (AHEI), Alternate Mediterranean Diet (aMED) and Dietary Approach to Stop Hypertension (DASH), during pregnancy and offspring weight outcomes [24]. An additional complexity in transgenerational studies concerns the careful consideration of the role of pregnancy-related variables and information collected throughout the offspring’s life. Particularly, pregnancy-related variables (e.g., maternal weight gain during pregnancy) could act as a confounding variable of the reported association. As we are linking information from two cohorts rather than analyzing data from a designed mother–child cohort, we only had limited information specific to the considered pregnancy. While offspring characteristics, such as dietary habits, reportedly contribute to the offspring obesity risk, conceptionally, they have a rather mediating role in our reported associations. Future analyses utilizing more detailed longitudinal information on offspring dietary patterns could help to disentangle these effects. Finally, weight outcomes in the offspring were based on self-reports with known limitations. If present, underreporting of weight by children who are overweight or obese may have contributed to an attenuation of the associations examined. Unique to our study is the use of cohort data with a long follow-up and detailed information on the dietary intake of mothers.

## 5. Conclusions

In conclusion, we found an association between a higher maternal phosphatidylcholine intake during pregnancy and offspring risk of being overweight or obese but not with maternal intakes of other one-carbon nutrients. Our findings warrant replication in other large transgenerational cohort studies with detailed dietary assessment of pregnant mothers.

## Figures and Tables

**Table 2:** Relative risks and 95% confidence intervals for offspring ever being overweight or obese during follow-up according to quintiles of maternal one-carbon nutrient intake during the period surrounding pregnancy among the n = 2434 offspring with at least one measurement of BMI.

	Quintile of Maternal One-Carbon Nutrient Intake					*p* for Trend
	1	2	3	4	5	
**Total folate**						
Median intake (µg/d)	275	439	708	1010	1355	
Cases/participants	231/483	235/489	224/488	222/487	223/488	
Basic model	1 (ref)	1.02 (0.90, 1.16)	0.96 (0.84, 1.10)	0.95 (0.83, 1.09)	0.96 (0.84, 1.10)	0.34
Multivariate model	1 (ref)	1.09 (0.96, 1.24)	1.06 (0.93, 1.21)	1.04 (0.91, 1.20)	1.03 (0.90, 1.18)	0.96
**Total vitamin B12**						
Median intake (µg/d)	5	8	11	14	20	
Cases/participants	250/534	246/525	213/459	207/449	219/468	
Basic model	1 (ref)	1.01 (0.89, 1.14)	1.00 (0.87, 1.14)	0.98 (0.86, 1.12)	1.00 (0.88, 1.15)	0.97
Multivariate model	1 (ref)	1.04 (0.92, 1.17)	1.07 (0.94, 1.22)	1.03 (0.90, 1.17)	1.04 (0.91, 1.19)	0.65
**Total vitamin B6**						
Median intake (mg/d)	2.0	3.0	4.3	6.4	12.6	
Cases/participants	228/485	248/505	219/478	224/482	216/485	
Basic model	1 (ref)	1.06 (0.93, 1.20)	0.97 (0.85, 1.11)	0.99 (0.86, 1.13)	0.96 (0.83, 1.10)	0.29
Multivariate model	1 (ref)	1.13 (0.99, 1.28)	1.04 (0.91, 1.19)	1.05 (0.92, 1.20)	1.02 (0.89, 1.17)	0.60
**Total vitamin B2**						
Median intake (mg/d)	1.8	2.6	3.6	4.4	5.9	
Cases/participants	233/482	225/487	229/492	223/484	225/490	
Basic model	1 (ref)	0.96 (0.84, 1.09)	0.95 (0.84, 1.09)	0.95 (0.84, 1.09)	0.95 (0.83, 1.08)	0.46
Multivariate model	1 (ref)	0.98 (0.86, 1.11)	1.03 (0.91, 1.17)	1.04 (0.91, 1.19)	1.01 (0.88, 1.15)	0.69
**Total methionine**						
Median intake (g/d)	1.6	1.9	2.1	2.2	2.5	
Cases/participants	211/488	213/474	242/495	237/497	232/481	
Basic model	1 (ref)	1.05 (0.91, 1.21)	1.13 (0.99, 1.30)	1.11 (0.97, 1.27)	1.11 (0.97, 1.28)	0.09
Multivariate model	1 (ref)	1.05 (0.92, 1.21)	1.12 (0.99, 1.28)	1.10 (0.96, 1.25)	1.07 (0.93, 1.23)	0.27
**Total choline**						
Median intake (mg/d)	269	311	338	366	407	
Cases/participants	200/484	227/490	251/486	231/486	226/488	
Basic model	1 (ref)	1.15 (1.00, 1.32)	1.25 (1.09, 1.43)	1.16 (1.01, 1.34)	1.14 (0.99, 1.32)	0.08
Multivariate model	1 (ref)	1.16 (1.01, 1.34)	1.26 (1.10, 1.44)	1.16 (1.01, 1.34)	1.16 (1.01, 1.34)	0.06
**Phosphatidylcholine**						
Median intake (mg/d)	115	139	158	176	206	
Cases/participants	204/487	205/486	225/490	248/487	253/485	
Basic model	1 (ref)	1.00 (0.87, 1.16)	1.10 (0.95, 1.26)	1.23 (1.07, 1.41)	1.26 (1.10, 1.44)	<0.0001
Multivariate model	1 (ref)	0.98 (0.85, 1.14)	1.06 (0.92, 1.22)	1.18 (1.03, 1.35)	1.16 (1.01, 1.33)	0.003
**Total betaine**						
Median intake (mg/d)	72	91	108	129	174	
Cases/participants	241/484	225/489	235/488	233/487	201/486	
Basic model	1 (ref)	0.94 (0.82, 1.07)	0.97 (0.86, 1.11)	0.97 (0.86, 1.11)	0.84 (0.73, 0.97)	0.03
Multivariate model	1 (ref)	0.97 (0.86, 1.10)	1.04 (0.91, 1.18)	1.06 (0.93, 1.20)	0.93 (0.81, 1.07)	0.46

Basic models are adjusted for offspring sex (boy/girl) and maternal age at birth of the child (continuous). Multivariable-adjusted models are additionally adjusted for BMI before pregnancy (<18.5, 18.5 < 25, 25–29, ≥30 kg/m^2^), smoking status before pregnancy (never, current, past), alcohol intake (g/d: 0, 1–14, or ≥15)), physical activity (0, 1–149, 150–299, ≥300 min/week of moderate to vigorous intensity), total energy intake (continuous), parity (nulliparous, 1, 2, 3+ previous pregnancies), partner’s education (less than 2 yr college, 4 yr college, graduate school), marital status (yes/no), sugar-sweetened beverages (servings/day in categories), refined grains (servings/day in categories), coffee (cups/day in quintiles), ratio of polyunsaturated to saturated fat (quintiles) and trans fat (grams per day in quintiles).

**Table 3:** Relative risks and 95% confidence intervals for offspring ever being overweight or obese during follow-up according to quintiles of maternal phosphatidylcholine intake during the period surrounding pregnancy stratified by major food sources.

	Quintiles of Maternal Phosphatidylcholine Intake					*p* for Trend	*p* for Interaction
	1	2	3	4	5		
**Egg**							
*Lower egg consumers*							
Median intake (mg/d)	113	139	156	174	204		
Cases/Offspring	129/312	92/211	73/155	70/136	37/78		
Basic model	1 (ref)	1.04 (0.85, 1.28)	1.14 (0.92, 1.41)	1.22 (0.99, 1.50)	1.15 (0.88, 1.49)	0.07	
Multivariable model	1 (ref)	1.02 (0.84, 1.25)	1.12 (0.91, 1.38)	1.19 (0.96, 1.46)	1.05 (0.80, 1.38)	0.25	
*Higher egg consumers*							
Median intake (mg/d)	120	140	158	176	207		
Cases/Offspring	75/175	113/275	152/335	178/351	216/407		
Basic model	1 (ref)	0.96 (0.77, 1.19)	1.06 (0.86, 1.30)	1.18 (0.97, 1.45)	1.24 (1.02, 1.51)	0.0007	
Multivariable model	1 (ref)	0.94 (0.76, 1.16)	1.02 (0.83, 1.25)	1.15 (0.95, 1.39)	1.14 (0.95, 1.38)	0.014	0.87
**Fish**							
*Lower fish consumers*							
Median intake (mg/d)	114	139	157	176	206		
Cases/Offspring	128/303	104/240	104/223	107/202	107/204		
Basic model	1 (ref)	1.03 (0.84, 1.25)	1.11 (0.92, 1.35)	1.26 (1.05, 1.51)	1.26 (1.04, 1.51)	0.003	
Multivariable model	1 (ref)	1.03 (0.85, 1.25)	1.09 (0.90, 1.32)	1.24 (1.03, 1.49)	1.18 (0.98, 1.43)	0.02	
*Higher fish consumers*							
Median intake (mg/d)	116	140	158	176	207		
Cases/Offspring	76/184	101/246	121/267	141/285	146/281		
Basic model	1 (ref)	0.99 (0.78, 1.24)	1.09 (0.87, 1.35)	1.19 (0.97, 1.46)	1.25 (1.02, 1.54)	0.004	
Multivariable model	1 (ref)	0.94 (0.75, 1.17)	1.02 (0.82, 1.26)	1.09 (0.89, 1.33)	1.10 (0.90, 1.35)	0.11	0.97
**Red meat**							
*Lower red meat consumers*							
Median intake (mg/d)	113	139	157	176	204		
Cases/Offspring	167/377	114/267	107/250	98/209	88/179		
Basic model	1 (ref)	0.96 (0.80, 1.15)	0.97 (0.81, 1.16)	1.05 (0.88, 1.26)	1.11 (0.92, 1.34)	0.23	
Multivariable model	1 (ref)	0.94 (0.78, 1.12)	0.94 (0.79, 1.13)	1.05 (0.88, 1.26)	1.05 (0.87, 1.27)	0.46	
*Higher red meat consumers*							
Median intake (mg/d)	120	140	158	176	207		
Cases/Offspring	37/110	91/219	118/240	150/278	165/306		
Basic model	1 (ref)	1.24 (0.91, 1.68)	1.47 (1.10, 1.97)	1.61 (1.21, 2.14)	1.62 (1.22, 2.15)	<0.0001	
Multivariable model	1 (ref)	1.23 (0.92, 1.65)	1.45 (1.10, 1.92)	1.58 (1.21, 2.08)	1.50 (1.14, 1.98)	0.001	0.13

Basic models are adjusted for offspring sex (boy/girl) and maternal age at birth of the child (continuous). Multivariable-adjusted models are additionally adjusted for BMI before pregnancy (<18.5, 18.5 < 25, 25–29, ≥30 kg/m^2^), smoking status before pregnancy (never, current, past), alcohol intake (g/d: 0, 1–14, or ≥15)), physical activity (0, 1–149, 150–299, ≥300 min/week of moderate to vigorous intensity), total energy intake (continuous), parity (nulliparous, 1, 2, 3+ previous pregnancies), partner’s education (less than 2 yr college, 4 yr college, graduate school), marital status (yes/no), sugar-sweetened beverages (servings/day in categories), refined grains (servings/day in categories), coffee (cups/day in quintiles), ratio of polyunsaturated to saturated fat (quintiles) and trans fat (grams per day in quintiles).

## Data Availability

The data underlying this article will be shared on reasonable request to the corresponding and last author.

## Supplementary Materials

The following supporting information can be downloaded at: https://www.mdpi.com/article/10.3390/nu16081210/s1: Supplementary Table S1. Adjusted mean differences (MDs) and 95% confidence intervals (95% CIs) in offspring birth weight (gram) according to quintiles of maternal one-carbon nutrient intake during the period surrounding pregnancy among the n = 1982 offspring with information on birth weight. Supplemental Table S2: Relative risks (RRs) for larger than median body size at age 5 according to quintiles of maternal one-carbon nutrient intake during the period surrounding pregnancy among the n = 2364 offspring with information on body size. Supplemental Table S3. Relative risks and 95% confidence intervals for offspring ever being overweight or obese during follow-up according to quintiles of maternal one-carbon nutrient intake during pregnancy among n = 896 mother–child pairs.
