# Supplementary material for: Maternal One-Carbon Nutrient Intake and Risk of Being Overweight or Obese in Their Offspring—A Transgenerational Prospective Cohort Study
Source: Nutrients. 2024 Apr 19;16(8):1210. doi: 10.3390/nu16081210 (PMC11054902; doi:10.3390/nu16081210)
Supplement: Supplementary file 1 [file nutrients-16-01210-s001.zip › nutrients-2956823-supplementary.pdf]

Supplemental Material to Bogl and Strohmaier et al. Maternal one-carbon nutrient intake and offspring overweight and obesity in their offspring – A transgenerational prospective cohort study

Supplementary Table S1. Adjusted mean differences (MD) and 95 % confidence intervals (95% CI) in offspring birth weight (gram) according to quintiles of maternal one-carbon nutrient intake during the period surrounding pregnancy among the n=1,982 offspring with information on birth weight

	Quintiles of maternal one-carbon nutrient intake					
	1	2	3	4	5	***P* for trend**
**Total folate**						
Participants	392	399	403	400	388	
Basic model, MD (95 % CI)	0 (ref)	-29.3 (-95.4, 36.8)	-18.4 (-84.2, 47.4)	33.5 (-32.5, 99.5)	-3.1 (-69.6, 63.4)	0.41
Multivariate model, MD (95 % CI)	0 (ref)	-23.9 (-92.0, 44.2)	-10.5 (-78.3, 57.3)	49.8 (-18.8, 118.4)	13.2 (-56.7, 83.1)	0.18
**Total vitamin B12**						
Participants	447	431	371	370	363	
Basic model, MD (95 % CI)	0 (ref)	-17.2 (-79.9,45.5)	-21.3 (-86.5, 43.9)	1.3 (-64.0, 66.5)	39.7 (-25.9, 105.3)	0.16
Multivariate model, MD (95 % CI)	0 (ref)	-20.8 (-84.2, 42.7)	-17.5 (-84.2, 49.1)	2.2 (-64.6, 69.0)	48.4 (-19.4, 116.2)	0.09
**Total vitamin B6**						
Participants	402	404	394	401	381	
Basic model, MD (95 % CI)	0 (ref)	-8.0 (-73.4,1, 57.4)	25.3 (-40.5, 91.1)	52.2 (-13.4, 117.7)	32.6 (-33.8, 98.9)	0.21
Multivariate model, MD (95 % CI)	0 (ref)	2.6 (-64.6, 69.9)	36.6 (-31.0, 104.2)	71.9 (4.2, 139.6)	46.4 (-22.0, 114.8)	0.14
**Total vitamin B2**						
Participants	396	396	407	392	391	
Basic model, MD (95 % CI)	0 (ref)	-54.5 (-120.4, 11.4)	15.0 (-50.4, 80.5)	41.6 (-24.5, 107.6)	25.7 (-40.4, 91.8)	0.06
Multivariate model, MD (95 % CI)	0 (ref)	-59.4 (-126.4, 7.6)	21.0 (-46.3, 88.3)	57.0 (-11.3, 125.3)	38.9 (-30.1, 107.9)	0.02
**Total methionine**						
Participants	396	383	417	390	396	
Basic model, MD (95 % CI)	0 (ref)	49.0 (-17.7, 115.6)	23.1 (-42.2, 88.2)	7.0 (-59.2, 73.2)	49.5 (-16.5, 115.4)	0.35
Multivariate model, MD (95 % CI)	0 (ref)	40.3 (-27.2, 107.8)	13.6 (-52.7, 79.9)	-4.0 (-71.5, 63.6)	36.4 (-32.2, 105.0)	0.60
**Total choline**						
Participants	399	396	396	398	393	
Basic model, MD (95 % CI)	0 (ref)	84.6 (18.8, 150.3)	28.0 (-37.7, 93.6)	71.2 (5.5, 136.9)	108.3 (42.4, 174.1)	0.005

Multivariate model, MD (95 % CI)	0 (ref)	81.7 (15.3, 148.1)	32.1 (-34.9, 99.1)	66.6 (-1.0, 134.1)	116.0 (47.1, 184.9)	0.004
**Phosphatidylcholine**						
Participants	400	401	392	395	394	
Basic model, MD (95 % CI)	0 (ref)	-31.4 (-97.0, 34.2)	-4.1 (-70.0, 61.9)	10.9 (-54.9, 76.7)	28.0 (-37.9, 93.9)	0.20
Multivariate model, MD (95 % CI)	0 (ref)	-29.0 (-95.1, 37.2)	-4.6 (-71.8, 62.7)	-0.9 (-67.9, 66.1)	17.2 (-50.2, 84.7)	0.42
**Total betaine**						
Participants	381	403	400	409	389	
Basic model, MD (95 % CI)	0 (ref)	-9.1 (-75.8, 57.5)	22.3 (-44.4, 88.9)	2.7 (-63.9, 69.2)	-37.9 (-105.4, 29.6)	0.27
Multivariate model, MD (95 % CI)	0 (ref)	-14.5 (-82.4, 53.4)	27.5 (-40.9, 96.0)	17.5 (-51.8, 86.8)	-12.6 (-83.2, 58.1)	0.87

Basic models are adjusted for offspring sex (boy/girl) and maternal age at birth of the child (continous).
Multivariable adjusted models are additionaly adjusted for BMI before pregnancy (< 18.5, 18,.5 < 25, 25-29, ≥ 30 kg/m2), smoking status before pregnancy (never, current, past), alcohol intake (g/d: 0, 1–14, or ≥ 15)), physical activity (0, 1-149, 150 -299, ≥ 300 min/week of moderate to vigorous intensity), total energy intake (continuous), parity (nulliparous, 1, 2, 3+ previous pregnancies), partner's education (less than 2yr college, 4yr college, graduate school), marital status (yes/no), sugar sweetened beverages (servings/day in categories), refined grains (servings/day in categories), coffee (cups/day in quintiles), ratio of polyunsaturated to saturated fat (quintiles) and trans fat (grams per day in quintiles).

Supplemental Table S2: Relative risks (RR) for larger than median body size at age 5 according to quintiles of maternal one-carbon nutrient intake during the period surrounding pregnancy among the n=2,364 offspring with information on body size.

	Quintiles of maternal one-carbon nutrient intake					
	1	2	3	4	5	***P* for trend**
**Total folate**						
Cases/participants	186/474	190/474	195/475	198/475	185/466	
Basic model	1 (ref)	1.03 (0.88, 1.20)	1.05 (0.90, 1.23)	1.06 (0.91, 1.24)	1.02 (0.87, 1.19)	0.76
Multivariate model	1 (ref)	1.06 (0.91, 1.25)	1.10 (0.94, 1.29)	1.10 (0.94, 1.29)	1.05 (0.89, 1.24)	0.55
**Total vitamin B12**						
Cases/participants	203/517	214/520	184/436	171/437	182/454	
Basic model	1 (ref)	1.06 (0.92, 1.23)	1.09 (0.93, 1.27)	1.01 (0.86, 1.18)	1.03 (0.88, 1.20)	0.99
Multivariate model	1 (ref)	1.07 (0.93, 1.25)	1.10 (0.94, 1.29)	1.01 (0.86, 1.18)	1.04 (0.89, 1.22)	0.96
**Total vitamin B6**						
Cases/participants	188/477	204/486	176/466	196/469	190/466	
Basic model	1 (ref)	1.07 (0.92, 1.24)	0.96 (0.82, 1.13)	1.07 (0.91, 1.24)	1.04 (0.89, 1.22)	0.69
Multivariate model	1 (ref)	1.11 (0.95, 1.30)	1.00 (0.85, 1.18)	1.09 (0.93, 1.27)	1.07 (0.91, 1.26)	0.60
**Total vitamin B2**						
Cases/participants	188/471	189/475	194/477	202/472	181/469	
Basic model	1 (ref)	1.01 (0.87, 1.18)	1.02 (0.87, 1.19)	1.08 (0.93, 1.26)	0.97 (0.83, 1.14)	0.90
Multivariate model	1 (ref)	1.02 (0.87, 1.19)	1.04 (0.89, 1.22)	1.09 (0.93, 1.27)	0.99 (0.84, 1.17)	0.88
**Total methionine**						
Cases/participants	202/471	183/459	193/476	197/486	179/472	
Basic model	1 (ref)	0.94 (0.81, 1.10)	0.96 (0.83, 1.11)	0.95 (0.82, 1.10)	0.88 (0.76, 1.03)	0.15
Multivariate model	1 (ref)	0.92 (0.79, 1.07)	0.93 (0.80, 1.08)	0.93 (0.80, 1.08)	0.85 (0.73, 1.00)	0.08
**Total choline**						
Cases/participants	182/467	195/476	207/473	192/473	178/475	
Basic model	1 (ref)	1.07 (0.92, 1.25)	1.12 (0.96, 1.30)	1.05 (0.90, 1.23)	0.97 (0.82, 1.14)	0.64
Multivariate model	1 (ref)	1.06 (0.92, 1.25)	1.11 (0.95, 1.30)	1.05 (0.89, 1.23)	0.96 (0.81, 1.13)	0.60
**Phosphatidylcholine**						
Participants	179/467	193/471	195/480	194/476	193/470	
Basic model	1 (ref)	1.07 (0.91, 1.25)	1.06 (0.91, 1.24)	1.07 (0.91, 1.25)	1.08 (0.92, 1.26)	0.40
Multivariate model	1 (ref)	1.05 (0.90, 1.23)	1.05 (0.90, 1.23)	1.05 (0.90, 1.24)	1.05 (0.90, 1.24)	0.56
**Total betaine**						
Cases/participants	188/475	196/474	207/473	190/472	173/470	
Basic model	1 (ref)	1.05 (0.90, 1.22)	1.10 (0.95, 1.28)	1.01 (0.87, 1.18)	0.93 (0.79, 1.10)	0.21
Multivariate model	1 (ref)	1.07 (0.92, 1.25)	1.13 (0.97, 1.32)	1.05 (0.90, 1.23)	0.97 (0.82, 1.15)	0.45

Basic models are adjusted for offspring sex (boy/girl) and maternal age at birth of the child (continous).
Multivariable adjusted models are additionaly adjusted for BMI before pregnancy (< 18.5, 18,.5 < 25, 25-29, ≥ 30 kg/m2), smoking status before pregnancy (never, current, past), alcohol intake (g/d: 0, 1–14, or ≥ 15)), physical activity (0, 1-149, 150 -299, ≥ 300 min/week of moderate to vigorous intensity), total energy intake (continuous), parity (nulliparous, 1, 2, 3+ previous pregnancies), partner’s education (less than 2yr college, 4yr college, graduate school), marital status (yes/no), sugar sweetened beverages (servings/day in categories), refined grains (servings/day in categories), coffee (cups/day in quintiles), ratio of polyunsaturated to saturated fat (quintiles) and trans fat (grams per day in quintiles).

Supplemental Table S3. Relative risks and 95% confidence intervals for offspring ever having overweight or obesity during follow-up according to quintiles of maternal one-carbon nutrient intake during pregnancy among n=896 mother child pairs

	Quintile of maternal one-carbon nutrient intake					
	1	2	3	4	5	***P* for trend**
**Total folate**						
Median intake (µg/d)	409	762	1000	1206	1531	
Cases/participants	92/178	84/179	81/180	87/179	83/180	
Basic model	1 (ref)	0.91 (0.74, 1.13)	0.87 (0.70, 1.08)	0.93 (0.76, 1.14)	0.91 (0.74, 1.13)	0.56
Multivariate model	1 (ref)	0.88 (0.72, 1.09)	0.86 (0.69, 1.07)	0.92 (0.75, 1.13)	0.90 (0.72, 1.12)	0.52
**Total vitamin B12**						
Median intake (µg/d)	7	9	13	16	21	
Cases/participants	97/197	66/132	99/218	89/187	76/162	
Basic model	1 (ref)	1.04 (0.83, 1.29)	0.94 (0.76, 1.15)	0.98 (0.80, 1.20)	0.97 (0.78, 1.21)	0.71
Multivariate model	1 (ref)	1.12 (0.90, 1.40)	0.98 (0.80, 1.19)	0.99 (0.81, 1.21)	1.00 (0.80, 1.25)	0.74
**Total vitamin B6**						
Median intake (mg/d)	2.7	4.4	5.7	9.8	15.5	
Cases/participants	94/176	82/183	81/179	81/179	89/179	
Basic model	1 (ref)	0.84 (0.68, 1.04)	0.84 (0.68, 1.05)	0.86 (0.69, 1.06)	0.95 (0.77, 1.16)	0.79
Multivariate model	1 (ref)	0.83 (0.68, 1.03)	0.83 (0.67, 1.02)	0.84 (0.68, 1.04)	0.92 (0.75, 1.13)	0.98
**Total vitamin B2**						
Median intake (mg/d)	2.4	3.6	4.2	4.9	6.2	
Cases/participants	84/175	92/182	89/180	76/180	86/179	
Basic model	1 (ref)	1.06 (0.86, 1.30)	1.04 (0.84, 1.28)	0.89 (0.71, 1.12)	1.01 (0.82, 1.26)	0.65
Multivariate model	1 (ref)	1.05 (0.86, 1.29)	1.03 (0.84, 1.27)	0.91 (0.72, 1.14)	1.04 (0.83, 1.30)	0.89
**Total methionine**						
Median intake (g/d)	1.6	1.9	2.1	2.2	2.5	
Cases/participants	76/180	80/172	91/187	93/181	87/176	
Basic model	1 (ref)	1.11 (0.88, 1.39)	1.16 (0.93, 1.45)	1.21 (0.97, 1.51)	1.17 (0.93, 1.46)	0.12
Multivariate model	1 (ref)	1.16 (0.93, 1.46)	1.23 (0.99, 1.52)	1.30 (1.05, 1.61)	1.18 (0.94, 1.48)	0.09
**Total choline**						
Median intake (mg/d)	267	310	339	367	404	
Cases/participants	79/178	89/181	83/179	91/180	85/178	
Basic model	1 (ref)	1.13 (0.91, 1.41)	1.05 (0.84, 1.32)	1.15 (0.92, 1.43)	1.09 (0.87, 1.36)	0.46
Multivariate model	1 (ref)	1.18 (0.95, 1.46)	1.11 (0.89, 1.39)	1.23 (0.99, 1.53)	1.14 (0.90, 1.43)	0.23
**Phosphatidylcholine**						
Median intake (mg/d)	115	138	157	175	207	

Cases/participants	76/179	86/180	79/180	92/180	94/177	
Basic model	1 (ref)	1.12 (0.89, 1.40)	1.04 (0.82, 1.31)	1.21 (0.97, 1.50)	1.26 (1.01, 1.57)	0.03
Multivariate model	1 (ref)	1.16 (0.93, 1.45)	1.05 (0.83, 1.32)	1.24 (1.00, 1.54)	1.18 (0.95, 1.46)	0.10
**Total betaine**						
Median intake (mg/d)	72	92	110	129	170	
Cases/participants	85/180	83/180	88/179	91/178	80/179	
Basic model	1 (ref)	0.98 (0.78, 1.22)	1.04 (0.84, 1.29)	1.08 (0.88, 1.34)	0.95 (0.76, 1.19)	0.83
Multivariate model	1 (ref)	1.02 (0.82, 1.27)	1.13 (0.91, 1.40)	1.24 (1.00, 1.53)	1.05 (0.83, 1.32)	0.52

Basic models are adjusted for offspring sex (boy/girl), maternal age at birth of the child (continous) and gestational age (28 - 36, 37 - 41, ≥ 42 wks). Multivariable adjusted models are additionaly adjusted for BMI before pregnancy (< 18.5, 18,.5 < 25, 25-29, ≥ 30 kg/m2), smoking status before pregnancy (never, current, past), alcohol intake (g/d: 0, 1–14, or ≥ 15)), physical activity (0, 1-149, 150 -299, ≥ 300 min/week of moderate to vigorous intensity), total energy intake (continuous), parity (nulliparous, 1, 2, 3+ previous pregnancies), partner's education (less than 2yr college, 4yr college, graduate school), marital status (yes/no), sugar sweetened beverages (servings/day in categories), refined grains (servings/day in categories), coffee (cups/day in quintiles), ratio of polyunsaturated to saturated fat (quintiles) and trans fat (grams per day in quintiles).
